# Quantum Interference Induced Photon Blockade in a Coupled Single Quantum Dot-Cavity System

**DOI:** 10.1038/srep09252

**Published:** 2015-03-18

**Authors:** Jing Tang, Weidong Geng, Xiulai Xu

**Affiliations:** 1 Institute of Photo-electronic Thin Film Devices and Technology, Nankai University, Tianjin 300071, P. R. China; 2 Beijing National Laboratory for Condensed Matter Physics, Institute of Physics, Chinese Academy of Sciences, Beijing 100190, P. R. China

## Abstract

We propose an experimental scheme to implement a strong photon blockade with a single quantum dot coupled to a nanocavity. The photon blockade effect can be tremendously enhanced by driving the cavity and the quantum dot simultaneously with two classical laser fields. This enhancement of photon blockade is ascribed to the quantum interference effect to avoid two-photon excitation of the cavity field. Comparing with Jaynes-Cummings model, the second-order correlation function at zero time delay *g*^(2)^(0) in our scheme can be reduced by two orders of magnitude and the system sustains a large intracavity photon number. A red (blue) cavity-light detuning asymmetry for photon quantum statistics with bunching or antibunching characteristics is also observed. The photon blockade effect has a controllable flexibility by tuning the relative phase between the two pumping laser fields and the Rabi coupling strength between the quantum dot and the pumping field. Moreover, the photon blockade scheme based on quantum interference mechanism does not require a strong coupling strength between the cavity and the quantum dot, even with the pure dephasing of the system. This simple proposal provides an effective way for potential applications in solid state quantum computation and quantum information processing.

Quantum information science (QIS) has been investigated intensively for their fascinating potential applications in quantum computation, cryptography, and metrology^1^,^2^,^3^,^4^,^5^. Among these applications, the realization of distribution, storage, and processing of quantum information in single-photon level^6^,^7^,^8^,^9^,^10^ are of great importance. Up to now, various platforms for implementing controllable single photons have been proposed, such as single atoms coupled with micro-cavity systems^11^,^12^,^13^,^14^, or single quantum dots integrated with photonic crystal cavities^15^,^16^,^17^,^18^,^19^, optical fibers^20^,^21^, and surface plasmons^22^.

A key point for single photon manipulation is to realize photon blockade. Photon blockade means that a first photon blocks the second photon transmission induced by the quantum anharmonicity ladder of energy spectrum with strong nonlinear interaction between single photons, corresponding to an orderly output of photons one by one with strong photon antibunching^23^. However, for a solid-state nanocavity with an embedded single quantum dot (QD), the strong coupling condition with 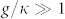 is hard to achieve due to the challenges of current micro-fabrication techniques for high-quality nanocavity^24^,^25^,^26^, where *g* is the QD-cavity coupling strength and *κ* is the cavity decay rate. To solve this problem, the photon blockade with strong sub-Poissonian light statistics based on bimodal-cavity scheme has been theoretically proposed^24^,^27^. Meanwhile, strong photon blockade can be obtained in photonic molecules with modest Kerr-nonlinearity of the photon using two coupled photonic cavities^28^,^29^,^30^,^31^. Unfortunately, the strong photon nonlinearity is very difficult to achieve at single photon level in most systems^32^, and the intracavity photon number is also low in the strong photon blockade regime.

In this paper, a novel scheme for generating a strong photon blockade with a single QD coupled to a nanocavity is proposed. Different from the Jaynes-Cummings (JC) model, our scheme requires an additional laser field to directly pump the single QD simultaneously. By utilizing the optimal quantum interference (QI) conditions, the cavity field exhibits the strong sub-Poissonian statistics and a red (blue) cavity-light detuning asymmetry, which is beyond the well known blockade mechanism induced by strong photon nonlinearity. More importantly, a large intracavity photon number (cavity output) is achieved with optimized parameters in photon blockade regime, even for a modest QD-cavity coupling strength. The *g*^(2)^(0) can be as low as 0.004 with a coupling strength of *g*/*κ* = 2. Consequently, it avoids the fabrication challenges for preparing nanocavities with high quality factors. Thus the proposed scheme can be used to obtain an ideal single photon source^33^, which is more feasible experimentally.

## Results

### Model and Hamiltonian

**Figure 1:**
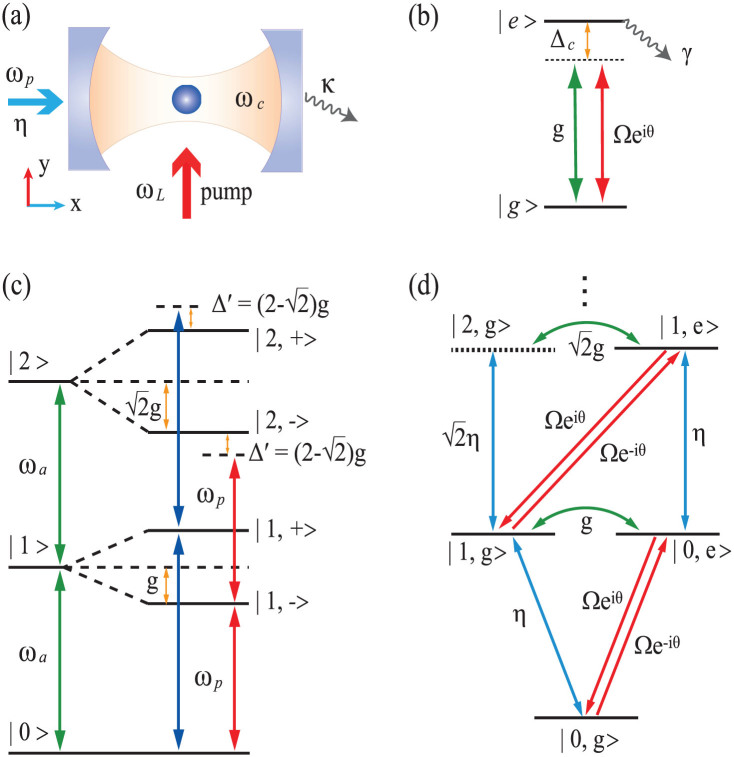
(a) Scheme for photon blockade of coupling a quantum dot with a nanocavity. (b) Level diagram for a quantum dot coupled with the cavity field and the pump field. (c) Energy level diagram of the dressed states in a coupled quantum dot-cavity system. (d) Transition paths for the Quantum Interference model.

We consider an excitonic two-level system of a single QD inside a nanocavity. As shown in Fig. 1(a), the single QD is coupled to the single mode nanocavity along *x* axis with a cavity frequency *ω_c_* and QD-cavity coupling strength *g*. The nanocavity is driven by a weak laser field with frequency *ω_p_*, coupling strength *η* and cavity decay rate *κ*. Additional pump field along *y* axis is applied to pump the single QD directly with a frequency of *ω_L_*, and provides a Rabi coupling strength Ω. Figure 1(b) shows the level structure for a single QD. In particular, even without cavity driven field, the excitonic |*g*〉 ↔ |*e*〉 transition with frequency *ω_a_* remains coupled to the nanocavtiy through the vacuum-stimulated Bragg scattering induced by the pump field^34^,^35^,^36^.

Using rotating wave approximation, the QD-cavity Hamiltonian can be described by (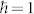)
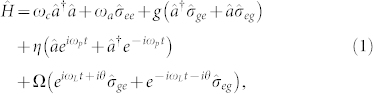
where 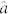 and 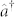 are the cavity mode annihilation and creation operators, 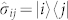 are the QD spin projection operators with *i*, *j* = *e*, *g* labeling the two involved levels, and *θ* is the relative phase between QD pumping field and cavity driven field.

For simplicity, we assume *ω_p_* = *ω_L_* and *ω_c_* = *ω_a_*. In the rotating frame with laser frequency *ω_p_* by utilizing the unitary transformation *U*,
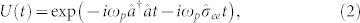
the interaction Hamiltonian of the QD-cavity system will be time-independent and can be rewritten as
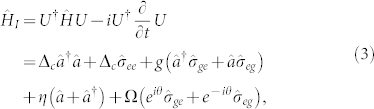
where Δ*_c_* = *ω_c_* − *ω_p_* = *ω_a_* − *ω_L_* is the cavity-light detuning. Similar to JC model, the new QI model with the Hamiltonian in Eq. (3) has an additional pump laser coupling the single QD directly. As discussed below, the relative phase *θ* of the two laser fields plays a significant role in the photon blockade effect.

Without the pump field for Ω = 0, the Hamiltonian in Eq. (3) is transformed to JC model. Neglecting the effect of weak driven field, the Hamiltonian can be exactly solved by projecting to a closed subspace with eigenstate basis |*n*, *g*〉 and |*n* − 1, *e*〉, where *n* is the number of photon excitation. Figure 1(c) shows the anharmonicity ladder of energy spectrum of JC model^37^, in which the dressed state |*n*, +(−)〉 represents the higher (lower) energy level of the *n*-th excited states with energy eigenvalues 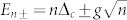, where 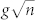 is the vacuum-Rabi splitting of the *n*-th excited states. When the first excited states are resonant with the laser field (Δ*_c_* = ±*g*), the energy levels of the second energy eigenstate |2, ±〉 are off-resonance with an energy gap of 
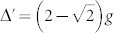
. In strong coupling limit 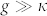, the process of two-photon excitation is strongly suppressed and photon blockade effect is enhanced with *g*^(2)^(0) ~ 0. It means a first photon “blocks” the second photon transmission to the cavity due to the far off-resonance two-photon absorption, where the second-order correlation function 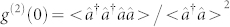 describes the quantum statistics of the photon field.

### Quantum interference mechanism

The photon blockade with anharmonic JC ladder is only achievable in a strong coupling regime, which is difficult to obtain in a single QD-nanocavity system. In our scheme, beyond the above photon blockade mechanism of anharmonic ladder with 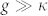, the strong photon blockade can be achieved even at a moderate QD-cavity coupling regime by simultaneously driving the cavity field and pumping the single QD as illustrated in Fig. 1(a). Since the applied pumping and driving fields are weak, the energy spectrum should be almost same with JC model as shown in Fig. 1(c). Because of the non-conserved excitation numbers, we cannot build a closed subspace with the *n*th block spanned by |*n* − 1, *e*〉 and |*n*, *g*〉. As a result, the Hamiltonian matrix can not be diagonalized exactly in the closed subspace. However, it can be diagonalized in the subspaces defined by a given excitation number of the cavity field. To understand the origin of the strong photon blockade, the wavefunction can be written as^29^
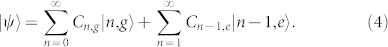
|*C_n_*_,*g*_|^2^ and |*C_n_*_−1,*e*_|^2^ represent the probabilities of eigenstates |*n*, *g*〉 and |*n* − 1, *e*〉, respectively. For the photon blockade case, we just need to cut off the photons into the two-photon excitation subspace with *n* = 2. So the wave function for the system can be expanded as: |*ψ*〉 = *C*_0,*g*_|0, *g*〉 + *C*_1,*g*_|1, *g*〉 + *C*_0,*e*_|0, *e*〉 + *C*_1,*e*_|1, *e*〉 + *C*_2,*g*_|2, *g*〉. To obtain the steady state solution, these probability coefficients are satisfying
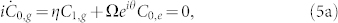

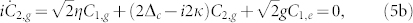

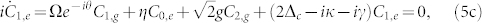
To suppress the two photon excitation, a condition *C*_2,*g*_ = 0 is required. In this limit, all higher photon excitations with *n* ≥ 2 are eliminated, resulting in only one excited photon in the nanocavity. It should be noted that this blockade mechanism is different from the strong coupling mechanism, where the higher photon excitations are far off-resonance due to anharmonicity of energy spectrum. The mechanism with strong photon blockade is ascribed to the quantum interference effect with different transition paths as shown in Fig. 1(d). Following the transition from |0, *g*〉 to |1, *g*〉 excited by the driven field, the interference can happen between the two paths, the direct transition 
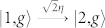
 and the transition 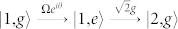. In absence of the pump field with Ω = 0, we can not find any non-trivial solution from Eqs. (5). When the pump field is applied, from the Eqs. (5) we can obtain the stead solution satisfying
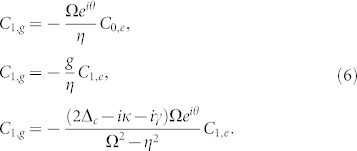


In vacuum-Rabi splitting with light-cavity detuning Δ*_c_* = ±*g*, the intracavity photon number should be large when the single photon level is excited resonantly. By solving Eq. (6) with Δ*_c_* = ±*g*, the optimized relative phase *θ*_o*pt*_ and Rabi coupling strength Ω_o*pt*_ are given by:
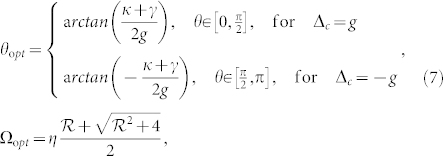
where 
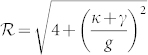
. The optimal parameter *θ*_o*pt*_ is dependent on the sign of Δ*_c_*, which means that photon blockade relies on the specific laser frequency. The optimal QI conditions in Eq (7) are the main results of this work.

Generally, when a cavity photon field has photon blockade effect, multiple photon excitations (*n* ≥ 2) are suppressed by strong photon nonlinearity or strong exciton-photon coupling. Here, however, the photon blockade can be realized with completely eliminating the two-photon excited states with *g*^(2)^(0) ~ 0 by using the QI mechanism with optimized conditions of Eq. (7), even for a moderate exciton-photon coupling strength. In the rest of the paper, we take nanocavity decay rate *κ*/2*π* = 20 GHz, single quantum dot spontaneous decay rate *γ*/2*π* = 1.0 GHz, and weak cavity driven strength *η* = 0.1*κ*.

### Numerical simulation

**Figure 2:**
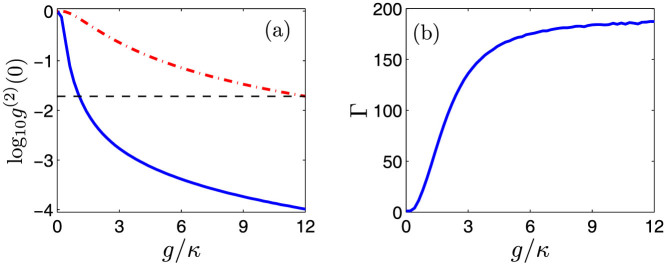
(a) The minimum second-order correlation function 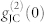 (dash-dotted red line) and 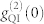 (solid blue line) as a function of the QD-cavity coupling strength *g*. (b) The ratio 
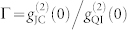
 is plotted as a function of the QD-cavity coupling strength *g*.

By solving the time dependent master equation (see Methods), the second-order correlation function *g*^(2)^(0) was calculated with (without) the laser for pumping the quantum dot in QI (JC) model. Figure 2(a) shows the minimum values of *g*^(2)^(0) for JC model with Ω = 0, and of *g*^(2)^(0) for QI model with (Ω, *θ*) = (Ω_opt_, *θ*_opt_) as a function of QD-cavity coupling strength *g*. Similar to the JC model, the second-order correlation function *g*^(2)^(0) monotonically decreases with increasing the coupling strength *g*, which suppresses the two-photon excitation due to a gradual increase of two-photon absorption energy gap Δ′. Surprisingly, photon blockade effect in the QI model is tremendously enhanced comparing with JC model at a specified coupling strength. For example, when log_10_*g*^(2)^(0) = −1.715 (as shown with the black-dashed line in Fig. 2(a)), the required coupling strength for JC model is *g*/*κ* = 12 while that for QI model is only 1.01. This indicates that a strong photon blockade can be achieved in a relative weak coupling strength in QI model.

It can be seen that the *g*^(2)^(0) of JC model quasi-linearly decreases as a function of coupling strength *g*. However, the *g*^(2)^(0) of QI model drops much more quickly, indicating that the *g*^(2)^(0) of QI model is more sensitive to *g*. To clearly show the difference between the two models, the ratio 
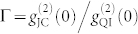
 as a function of coupling strength *g* is plotted in Fig. 2(b). At a strong coupling regime or even a moderate regime with *g*/*κ* > 2, the photon blockade for QI model is enhanced by two orders of magnitude. It shows that a strong photon anti-buntuning (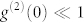) with sub-Poissonian quantum statistics for cavity field output can be easily achieved using the quantum interference method.

**Figure 3:**
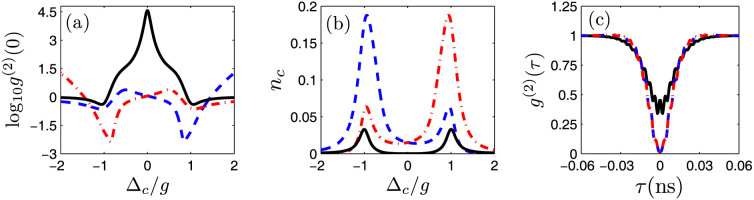
(a) The second-order correlation function *g*^(2)^(0) and (b) the mean cavity photon number *n_c_* as a function of cavity-light detuning Δ*_c_*. (c) The time-dependent second-order correlation function *g*^(2)^(*τ*) of the coupled system. The solid black lines show the results of JC model with Ω/*g* = 0. The dashed blue lines and dash-dotted red lines represent the results with (Ω/*g*, *θ*/*π*) = (0.124, 0.082) and (Ω/*g*, *θ*/*π*) = (0.124, ±1 − 0.082) in the QI model, respectively.

With a moderate QD-cavity coupling strength *g* = 2*κ*, the second-order correlation function *g*^(2)^(0) and the intracavity photon number *n_c_* as a function of cavity-light detuning Δ*_c_* are shown in Fig. 3(a) and 3(b), respectively. For JC model, both *g*^(2)^(0) and *n_c_* are symmetric for the red and blue detuning with Δ = ±*g*, and are phase-independent for the weak cavity driven field. However, asymmetric structures of QI model for both *g*^(2)^(0) and *n_c_* are observed. With an optimized phase condition tan *θ* = (*κ* + *γ*)/(2*g*) = 0.2625 and a red detuning Δ*_c_* = *g*, photon blockade can be observed at the position of Δ*_c_* ≈ *g* with *g*^(2)^(0) ≈ 0, indicating sub-Poissonian quantum statistics for cavity field. But at the position with Δ*_c_* ≈ −*g*, the *g*^(2)^(0) is close to unity, which is similar to the results in JC model (black solid line). A reversed result can be obtained with the optimized phase condition at blue detuning Δ*_c_* = −*g*, as shown by the dash-dotted red line in Fig. 3(a).

The minimum *g*^(2)^(0) is about 0.004 when the laser field is tuned to satisfy the optimized QI conditions of Eq. (7). Especially, even in the photon blockade regime, an intracavity photon number *n_c_* of about 0.06 is still larger than the maximum intracavity number in the JC model [as shown in Fig. 3(b)], which is a key factor for ideal single photon sources with a large cavity output^37^. Figure 3(c) shows *g*^(2)^(*τ*) as a function of time. Anti-bunching effect with *g*^(2)^(0) < 1 and *g*^(2)^(0) < *g*^(2)^(*τ*) indicates the output light is sub-Poissonian and antibunching^38^. The *g*^(2)^(*τ*) rises to unity at a time 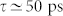, which is consistent with the lifetime *τ* = 1/(*γ* + *κ*) = 48 ps for the dressed states |1, ±〉^25^,^37^.

**Figure 4:**
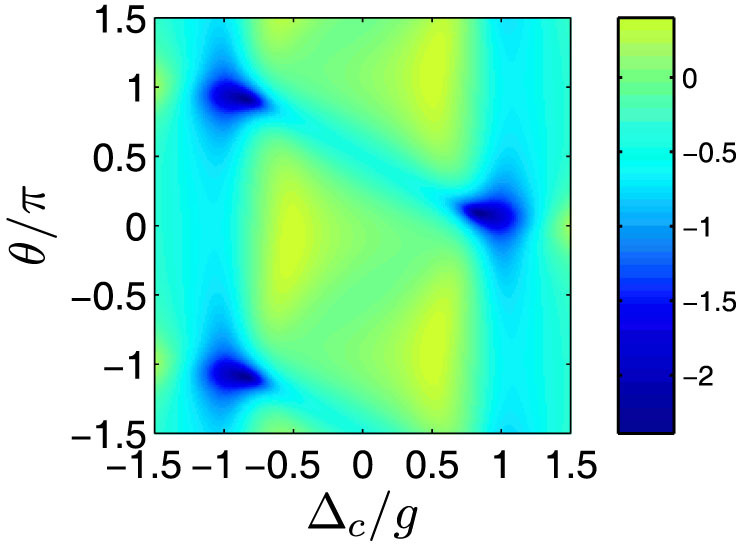
The second-order correlation function in logarithmic scale (log_10_*g*^(2)^(0)) as a function of cavity-light detuning Δ*_c_* and relative dynamic phase *θ* for *g* = 2*κ* and Ω/*g* = 0.124.

To further investigate the red-blue detuning asymmetry, we calculated *g*^(2)^(0) as a function of cavity-light detuning Δ*_c_* and relative phase *θ* with an optimized Rabi coupling strength Ω/*g* = 0.124. As illustrated in Fig. 4, a red (blue) detuning asymmetric feature for *g*^(2)^(0) is observed, which is strongly correlated to the relative phase *θ*. For example, with a phase of *θ*/*π* ≈ 0.08, tan *θ* > 0, the *g*^(2)^(0) at a red detuning position of Δ*_c_* ≈ *g* approaches its minimum, which exhibits a strong sub-Poissonian quantum statistics, whereas at the blue detuning the *g*^(2)^(0) is close to unity. Similar features can be observed with phases at −0.082*π* ± *π* for the blue detuning case with Δ*_c_* ≈ −*g*. Therefore in the QI model, the relative phase *θ* is non-trivial and significantly influences the cavity quantum statistics and output, which can not be eliminated by gauge transformation. The simulation results by solving master equation verify the theoretical prediction with optimized QI conditions in Eq. (7).

**Figure 5:**
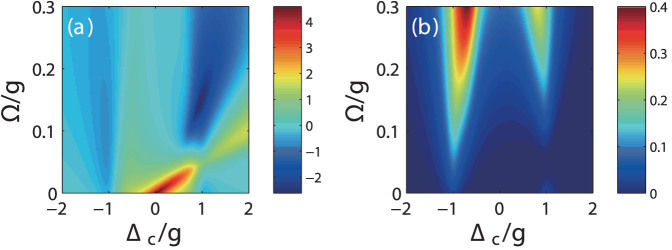
(a) The second-order correlation function in logarithmic scale (log_10_(*g*^(2)^(0))) and (b) the intracavity photon number *n_c_* as a function of cavity-light detuning Δ*_c_* and Rabi Rabi coupling strength Ω for *g* = 2*κ* and *θ*/*π* = 0.082.

Figure 5(a) and 5(b) show the contour plots of *g*^(2)^(0) and *n_c_* as a function of Δ*_c_* and Ω with a fixed phase *θ*_opt_/*π* = 0.082. As expected, a strong photon blockade should occur near the red detuning with Δ*_c_* ≈ *g*. While for blue detuning with Δ*_c_* ≈ −*g*, there is no strong blockade because the phase of 0.082*π* is not an optimized value in this case. Therefore, a higher intracavity photon number for blue detuning regime is expected as shown in Fig. 5(b). Note that at red detuning with Δ*_c_* ≈ *g*, intracavity photon number *n_c_* is still much larger than the mean photon number *n_c_* = (*η*/*κ*)^2^ = 0.01 in an empty cavity at strong photon blockade regime. This means that this scheme can achieve an ideal single photon source using solid-state single quantum dots with a strong photon blockade and a large cavity output. In fact, a moderate QD-cavity coupling strength *g* is sufficient for this purpose, which means that we do not need high quality factors (*Q*) for the nanocavities. In addition, the calculations show that photon blockade effect can survive with a relatively large parameter variation. As a result, the robustness of photon blockade for single QDs does not need to perfectly satisfy the optimal QI conditions in Eq. (7), which should be more easily to be achieved experimentally. In certain regimes, *g*^(2)^(0) with strong super-Poissonian quantum statistics is also observed for off-resonant excitation at Δ*_c_* ≈ 0.16*g* but with an ultra-low intracavity photon number *n_c_* = 1.613 × 10^−5^.

**Figure 6:**
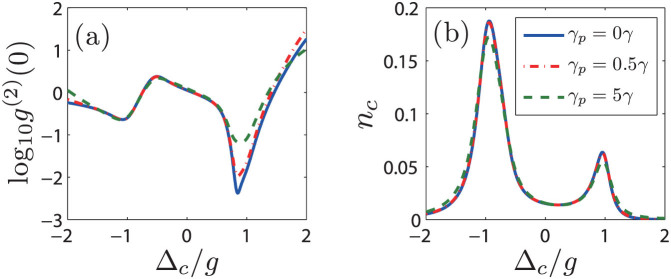
(a) The second-order correlation function in logarithmic scale (log_10_(*g*^(2)^(0))) and (b) the intracavity photon number *n_c_* as a function of cavity-light detuning Δ*_c_* with *g* = 2*κ* and (Ω/*g*, *θ*/*π*) = (0.124, 0.082) for different pure dephasing *γ_d_*. The solid blue line, the dash-dotted red line, and the dashed green line represent the results with *γ_d_* at 0*γ*, 0.5*γ*, and 5*γ*, respectively.

So far, we did not consider the effect of pure dephasing, which could affect the polarization^39^, linewidth^40^,^41^, photon statistics and cavity transmission^16^,^42^,^43^,^44^ in solid state QD-cavity systems. Next, we study the effect of pure dephasing *γ_d_* on photon blockade in the QI model by adding a Lindblad term 
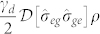
 in the master equation. Figure 6 shows the second-order correlation function *g*^(2)^(0) and the intracavity photon number *n_c_* with different pure dephasing rates. It can be seen that the *g*^(2)^(0) and *n_c_* still maintain the red-blue detuning asymmetry. With increasing the pure dephasing rate, *n_c_* does not change too much, but *g*^(2)^(0) increases near the red detuning with Δ*_c_* ≈ *g*, while remains the same at the blue detuning with Δ*_c_* ≈ −*g*. Nevertheless, the qualitative nature of the photon blockade is unchanged. For a typical pure dephasing rate *γ_d_* = 0.5*γ*^39^, the *g*^(2)^(0) at the red detuning with Δ*_c_* ≈ *g* is 0.01, and the corresponding *n_c_* is still large for a coupling strength *g* = 2*κ*, which still can be treated as an ideal single photon source with photon blockade.

## Discussion

We proposed a new QI model with a simple configuration by simultaneously driving the cavity field and the single QD and realized strong photon blockade in a QD-cavity system. Photon distributions with strongly antibunching effect and sub-Poissonian statistics have been observed by numerically solving the master equation using optimized phase *θ* and the coupling strength Ω. Furthermore, a red (blue) detuning asymmetry for photon blockade has been observed. Photon blockade with a large intracavity number for quantum dot shows a strong robustness, which can be easily realized experimentally with considering the pure dephasing. From a practical point of view, it might be not easy to excite quantum dot and cavity separately. However, several schemes have been demonstrated successfully by using different pumping pulse widths^19^, or by spatially/spectrally decoupling the driving fields for quantum dot and cavity^45^,^46^,^47^. We believe the proposed scheme with QI mechanism could be very helpful for applications in various cavity quantum-electrodynamics systems.

## Methods

In order to demonstrate the photon blockade, we investigated the quantum statistics of the nanocavity field by solving quantum master equation numerically. Considering the dissipation of the cavity with decay rate *κ* and QD spontaneous emission rate *γ*, without the nonradiative pure dephasing, the master equation of the dynamics of single QD-cavity system satisfies,
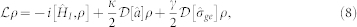
where *ρ* is density matrix of QD-cavity system, 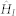 is the time-independent interaction Hamiltonian of Eq. (3), 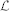 is Liouvillian superoperator, and 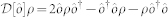 is the Lindblad type of dissipation. Then the steady state intracavity photon number 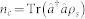 and the second-order correlation function 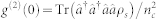 can be obtained by calculating the steady state density matrix with 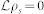 using Quantum Optics Toolbox^48^.

## Author Contributions

J.T. performed calculations. X.X. and W.G. supervised the project. J.T. and X.X. wrote the paper and all authors reviewed the manuscript.
